# Comprehensive Analysis of PD-L1 Expression, Immune Infiltrates, and m6A RNA Methylation Regulators in Esophageal Squamous Cell Carcinoma

**DOI:** 10.3389/fimmu.2021.669750

**Published:** 2021-05-12

**Authors:** Wei Guo, Fengwei Tan, Qilin Huai, Zhen Wang, Fei Shao, Guochao Zhang, Zhenlin Yang, Renda Li, Qi Xue, Shugeng Gao, Jie He

**Affiliations:** ^1^ Department of Thoracic Surgery, National Cancer Center/National Clinical Research Center for Cancer/Cancer Hospital, Chinese Academy of Medical Sciences and Peking Union Medical College, Beijing, China; ^2^ Department of Graduate School, Zunyi Medical University, Zunyi, China; ^3^ Cancer Institute of The Affiliated Hospital of Qingdao University, Qingdao Cancer Institute, Qingdao, Shandong, China

**Keywords:** esophageal squamous cell carcinoma, N6-methyladenosine methylation, RNA methylation, prognosis, immune infiltrates, PD-L1

## Abstract

**Background:**

Esophageal squamous cell carcinoma (ESCC) is one of the most common cancer types and represents a threat to global public health. N6-Methyladenosine (m6A) methylation plays a key role in the occurrence and development of many tumors, but there are still few studies investigating ESCC. This study attempts to construct a prognostic signature of ESCC based on m6A RNA methylation regulators and to explore the potential association of these regulators with the tumor immune microenvironment (TIME).

**Methods:**

The transcriptome sequencing data and clinical information of 20 m6A RNA methylation regulators in 453 patients with ESCC (The Cancer Genome Atlas [TCGA] cohort, n = 95; Gene Expression Omnibus [GEO] cohort, n = 358) were obtained. The differing expression levels of m6A regulators between ESCC and normal tissue were evaluated. Based on the expression of these regulators, consensus clustering was performed to investigate different ESCC clusters. PD-L1 expression, immune score, immune cell infiltration and potential mechanisms among different clusters were examined. LASSO Cox regression analysis was utilized to obtain a prognostic signature based on m6A RNA methylation modulators. The relationship between the risk score based on the prognostic signature and the TIME of ESCC patients was studied in detail.

**Results:**

Six m6A regulators (METTL3, WTAP, IGF2BP3, YTHDF1, HNRNPA2B1 and HNRNPC) were observed to be significantly highly expressed in ESCC tissues. Two molecular subtypes (clusters 1/2) were determined by consensus clustering of 20 m6A modulators. The expression level of PD-L1 in ESCC tissues increased significantly and was significantly negatively correlated with the expression levels of YTHDF2, METL14 and KIAA1429. The immune score, CD8 T cells, resting mast cells, and regulatory T cells (Tregs) in cluster 2 were significantly increased. Gene set enrichment analysis (GSEA) shows that this cluster involves multiple hallmark pathways. We constructed a five-gene prognostic signature based on m6A RNA methylation, and the risk score based on the prognostic signature was determined to be an independent prognostic indicator of ESCC. More importantly, the prognostic value of the prognostic signature was verified using another independent cohort. m6A regulators are related to TIME, and their copy-number alterations will dynamically affect the number of tumor-infiltrating immune cells.

**Conclusion:**

Our study established a strong prognostic signature based on m6A RNA methylation regulators; this signature was able to accurately predict the prognosis of ESCC patients. The m6A methylation regulator may be a key mediator of PD-L1 expression and immune cell infiltration and may strongly affect the TIME of ESCC.

## Introduction

Esophageal cancer (EC) is a highly aggressive malignancy and is the eighth most common malignant tumor in the world; furthermore, the mortality rate of EC ranks sixth worldwide and is still rising (1–3). As with the National Central Cancer Registry of China (NCCR) statistics, Chinese EC patients compose up to 70% of all EC cases worldwide (4). Among the two main histopathological subtypes of EC, esophageal squamous cell carcinoma (ESCC) is more common than esophageal adenocarcinoma (5). Neoadjuvant chemotherapy or chemoradiotherapy have been utilized as the standard treatments for ESCC, improving the surgical effect (6). Although related immunotherapies involving esophageal cancer are still in the preliminary stages of research, some related immunosuppressants have entered clinical trials and have exhibited long-lasting antitumor activity and controllable adverse reactions. Studies have found that PD-L1 positive patients with advanced and metastatic esophageal cancer are very likely to be sensitive to immunotherapy (7). Abnormal levels of N6-methyladenosine (m6A) methylation also play vital roles in the progression of various cancers (8). Accurately predicting the prognosis of ESCC is the key to the success of clinical diagnosis and treatment and individualized medication. Therefore, the identification of novel and reliable prognostic molecular signatures from multiple dimensions is very important for selecting the most appropriate treatment strategy and improving the poor prognosis of ESCC patients.

Numerous studies in recent years have shown that N6-methyladenosine (m6A) methylation is a commonly seen modification in eukaryotic messenger RNA (mRNA) and strongly affects many basic biological processes, such as cell differentiation, tissue development and tumorigenesis (9–12). The level of m6A methylation is regulated by methyltransferases, demethylases, and binding proteins. m6A RNA modification can be catalysed enzymatically by various methyltransferases, known as m6A “writers” (METTL3, METTL14, METTL16, KIAA1429, WTAP, RBM15, RBM15B and ZC3H13). N6-methyladenosine in RNA can be removed by demethylases, known as m6A “erasers” (FTO and ALKBH5). Proteins that selectively bind m6A can be defined as m6A “readers” (YTHDC1, YTHDC2, IGF2BP2, IGF2BP3, YTHDF1, YTHDF2, YTHDF3, HNRNPA2B1, HNRNPC, and RBMX) that exert regulatory functions by selective recognition of methylated RNA (13). In addition, emerging evidence suggests that m6A modulators have cancer promoter or inhibitory effects in the development of various malignant tumors. Zhang et al. demonstrated the m6A modulator-mediated methylation modification pattern and the tumor microenvironment infiltration characteristics of gastric cancer (14–16). Han et al. (17) confirmed that m6A methylation can prolong the neoantigen-specific immunity mediated by YTHDF1. YTHDF1 may be a potential therapeutic target and an important mediator of tumor immune evasion. These findings suggest that both m6A methylation regulators and the tumor immune microenvironment (TIME) may affect the prognosis of cancer patients. The TIME may affect the patient’s response to immune checkpoint inhibitors, so PD-L1 expression in ESCC should be further considered to evaluate tumor immunity. However, to the best of our knowledge, there are few studies on the use of m6A methylation regulators to predict the prognosis of ESCC. In addition, the correlation between m6A methylation regulators and PD-L1, the expression of PD-L1 in ESCC, and the abundance of immune infiltrating cells need to be fully studied.

In this study, RNA sequencing data, clinical information and immune cell data of ESCC patients were obtained from The Cancer Genome Atlas (TCGA). According to the expression of m6A RNA methylation regulators, consensus cluster analysis was performed, and a gene signature and risk model were constructed to predict the prognosis of ESCC patients more accurately. Although there have been reports of consensus cluster analysis on patients with esophageal cancer (18), the study did not separate the subgroups of esophageal squamous cell carcinoma and further verification, and the number of m6A RNA methylation regulators included in the study was not large enough. To enhance the prediction performance of the m6A methylation-related gene signature, we verified it in another ESCC cohort in the Gene Expression Omnibus (GEO) database. In addition, we also studied the infiltration of immune cells in the TIME, the expression of PD-L1 in ESCC, and the correlation with m6A RNA methylation regulators.

## Materials and Methods

### Data Acquisition

Clinical information, including sex, T stage, N stage, M stage, TNM stage, survival information and RNA-seq expression profiles from ESCC (95 patients) cohorts of TCGA database (https://portal.gdc.cancer.gov), was used in this study. We also selected gene expression profiles of patient-derived ESCC tissue (GSE53625, GPL18109, n = 358) from the GEO database to validate the candidate prognostic gene signature identified from TCGA data. This data set contains gene sequencing information and clinical information of cancer tissues and adjacent normal tissues from 358 ESCC patients in China.

### m6A RNA Methylation Regulator Collection

According to previously published research reports, we collected 20 m6A RNA methylation regulators (METTL3, METTL14, METTL16, KIAA1429, WTAP, RBM15, RBM15B, ZC3H13, YTHDC1, YTHDC2, IGF2BP2, IGF2BP3, YTHDF1, YTHDF2, YTHDF3, HNRNPA2B1, HNRNPC, RBMX, FTO and ALKBH5) for further research.

### Bioinformatic Analysis

The Search Tool for the Retrieval of Interacting Genes/Proteins database (STRING, version 11.0, http://string-db.org/) has powerful functions in studying gene interactions and visualization (19). We entered 20 m6A RNA methylation regulators into STRING to understand their interactions and performed functional annotation analysis to initially explore the biological process (BP), cell composition (CC), molecular function (MF) and Kyoto Encyclopedia of Genes and Genomes (KEGG) pathways involved in these regulators. Pearson correlation analysis was used to elucidate the correlation between different m6A RNA methylation regulators.

To functionally explore the biological properties of m6A regulators in ESCC, we used the “ConsensusClusterPlus” package (http://www.bioconductor.org/, 1000 iterations and resampling rate of 80%) to divide ESCC patients from the TCGA database into different groups. Gene set enrichment analysis (GSEA) was utilized to understand the biological processes involved in different subgroups. Hallmarks in GSEA were used to identify predefined gene sets; 5000 permutations were performed according to the gene set to determine p-values. A pathway with a p-value < 0.01 and a false discovery rate (FDR) < 0.25 was considered to be significant, as described in the Results section.

CIBERSORT (http://cibersort.stanford.edu/), a deconvolution algorithm based on gene expression, can calculate the composition of immune cells from the gene expression profile of complex tissues (20). We used CIBERSORT software to calculate the infiltration level of 22 immune cells based on the expression profile data of ESCC in the TCGA database. Subsequently, the ESTIMATE algorithm (“estimate” package in R) was used to calculate the immune score of each patient, and the difference in the immune score between the two cluster subgroups was evaluated (21).

The least absolute shrinkage and selection operator (LASSO) Cox regression model includes all m6A methylation regulators to construct a strong prognostic signature and calculate the coefficient of each gene. The coefficients obtained from the LASSO regression algorithm were used to yield the following risk score equation: risk score = sum of coefficients × m6A regulator expression level. According to the score, the gene signature with the strongest ability to predict the prognosis of ESCC patients was obtained. The ESCC patients were divided into high- and low-risk groups according to the median risk score, and the difference in overall survival (OS) between the risk score groups was evaluated. Through the receiver operating characteristic (ROC) curve and the area under the ROC curve (AUC value), the accuracy of the genetic signature for predicting prognosis was evaluated. Univariate and multivariate Cox regression analyses were used to identify independent prognostic factors for ESCC patients. Importantly, we verified this prognostic signature using a cohort of 358 ESCC samples from the GEO database.

Tumor Immune Estimation Resource (TIMER, https://cistrome.shinyapps.io/timer/) was used to analyze the six main immune cells in tumors to evaluate the effect of copy number alternations (CNAs) of m6A regulators on the level of immune cell infiltration.

### Statistical Analysis

R (version 3.6.3; The R Foundation for Statistical Computing) and SPSS 23.0 (IBM Corp., New York, USA) were used for data analysis and statistics. m6A regulator expression and clinical data were analysed by χ^2^ test or Fisher’s exact test. Student’s t-test and one-way ANOVA were used to separately perform the group comparisons of two subgroups and more than two subgroups. Kaplan-Meier survival analysis and log-rank tests were used to analyse the differences in OS between different risk score groups. The correlation of gene expression was evaluated by Spearman’s R and statistical significance. An absolute value of R greater than 0.25 was considered relevant, and a p-value < 0.05 was considered to be significant.

## Results

### Expression of m6A RNA Methylation Regulators in ESCC

**Figure 1:**
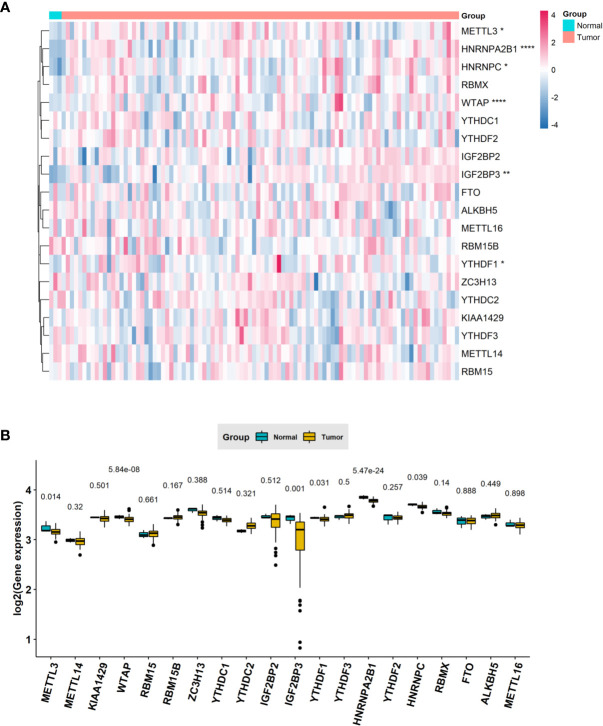
The expression levels of m^6^A RNA methylation regulators between tumor and normal samples in TCGA ESCC cohort. **(A)** Heatmap of m^6^A RNA methylation regulator expression level in each sample; **(B)** the expression difference of m6A RNA methylation regulator between tumor and normal samples. * means p<0.05; ** means p<0.01; **** means p<0.0001.

To understand the expression pattern of m6A RNA methylation regulators between ESCC tumors and normal tissues, we drew a heatmap based on 20 m6A methylation genes in the TCGA database. The red or green in the figure indicates relatively high or low expression, respectively (**Figure 1A**). The expression levels of writers (METTL3 and WTAP) and readers (i.e., IGF2BP3, YTHDF1, HNRNPA2B1 and HNRNPC) were notably higher in ESCC tissues than in normal adjacent tissues (**Figure 1B**) (*P* < 0.05). The abnormal expression of these m6A RNA methylation regulators may indicate an important biological role in the occurrence and development of ESCC.

### Correlation and Functional Enrichment Between m6A RNA Methylation Regulators

**Figure 2:**
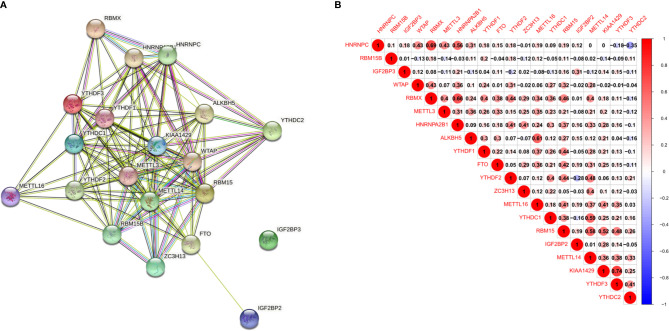
Interaction and correlation between m^6^A RNA methylation regulators in ESCC. **(A)** A PPI network was constructed to evaluate the interaction between m^6^A RNA methylation regulators; **(B)** the correlations among m^6^A RNA methylation regulators were analyzed by Pearson correlation.

With the STRING database used to further understand the interaction between the 20 m6A RNA methylation regulators, a PPI network was obtained. After deleting the isolated genes without interaction, we determined that the PPI network contained 20 nodes and 112 edges, as shown in **Figure 2A**. KIAA1429 and METTL3 appeared to be the hub genes of the interaction network. As shown in **Table 1**, we performed Gene Ontology (GO) analysis on these 20 RNA methylation regulators to obtain a preliminary understanding of their biological functions. Regulation of mRNA metabolic process, N6-methyladenosine-containing RNA binding and RNA N6-methyladenosine methyltransferase complex were the most significantly enriched GO items. In addition, we observed that all m6A RNA methylation regulators were generally positively correlated, and KIAA1429 had the highest correlation with YTHDF3 (**Figure 2B**) (r = 0.74).

**Table 1:** Functional annotation of 20 m6A methylation regulators.

Category	GO-term	Description	Gene count	*P*-value
BP	GO:1903311	Regulation of mRNA metabolic process	15	2.43E-22
BP	GO:0080009	mRNA methylation	6	1.47E-12
BP	GO:0098508	Endothelial to hematopoietic transition	2	5.27E-05
BP	GO:0035553	Oxidative single-stranded RNA demethylation	2	7.66E-05
MF	GO:1990247	N6-methyladenosine-containing RNA binding	7	1.23E-16
MF	GO:0003723	RNA binding	15	2.71E-15
MF	GO:0003729	mRNA binding	8	5.02E-10
MF	GO:0003730	mRNA 3’-UTR binding	4	8.49E-06
CC	GO:0036396	RNA N6- methyladenosine methyltransferase complex	7	1.47E-16
CC	GO:0016607	Nuclear speck	9	1.01E-09
CC	GO:0016604	Nuclear body	10	1.11E-08
CC	GO:1902494	Catalytic complex	10	1.80E-06

BP, Biological process; MF, Molecular function; CC, Cellular components.

### Consensus Clustering Identified Two Clusters of Patients With ESCC

**Figure 3:**
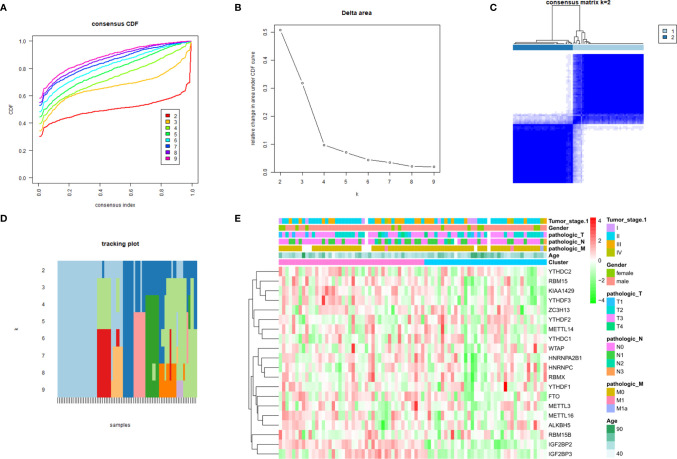
Consensus clustering identified two ESCC patient clusters and their relationship with clinicopathological parameters. **(A)** Consensus clustering cumulative distribution function (CDF) for k=2 to 9; **(B)** relative change in area under CDF curve for k=2 to 9; **(C)** The ESCC cohort from TCGA was divided into two distinct clusters when k=2; **(D)** distribution of each sample when k ranges from 2 to 9; **(E)** comparison of the relationship between the clinicopathological characteristics of two clusters.

A consensus cluster consisting of 20 m6A RNA methylation regulators was constructed by using the “ConsensusClusterPlus” package. **Figures 3A, B** show the relative change of the cumulative distribution function (CDF) of the consensus cluster from k = 2 to 9 and the area under the CDF curve from k = 2 to 9, and k = 2 is proven to be the most suitable choice to divide the ESCC patient cohort into two clusters (**Figure 3C**). The tracking plot for k = 2 to k = 10 is demonstrated in **Figure 3D**. In addition, we studied the relationship between clustering subgroups and clinicopathological parameters of ESCC patients. The results showed that there was no significance between cluster 1 and cluster 2 and patient age, sex, T stage, N stage, M stage, or TNM stage (**Figure 3E**).

### Association of PD-L1 With m6A RNA Methylation

**Figure 4:**
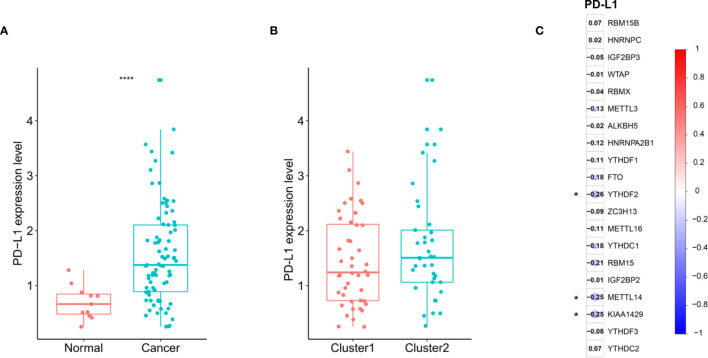
Association of PD-L1 with m6A RNA methylation and the landscape of immune cell infiltration in ESCC. **(A)** PD-L1 expression was significantly higher in ESCC cohort from TCGA; **(B)** the expression level of PD-L1 in cluster1/2 subtypes; **(C)** the correlation of PD-L1 with m^6^A methylation regulators in ESCC cohort from TCGA. **** means p<0.0001.

**Figure 5:**
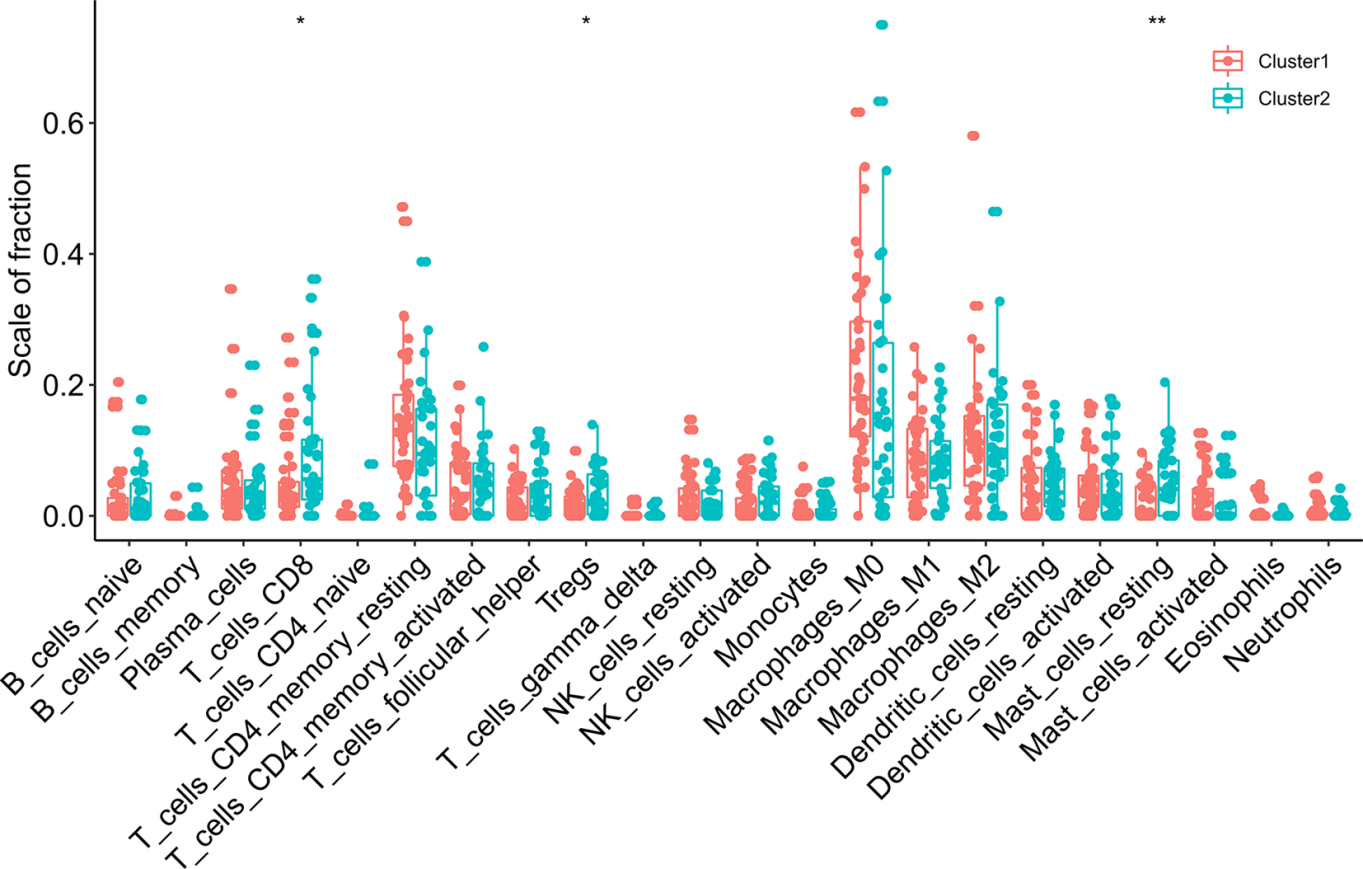
The infiltrating levels of 22 immune cell types in cluster1/2 in ESCC. * means p<0.05; ** means p<0.01.

We assessed the difference in PD-L1 expression between tumor and normal tissues in ESCC patients. Compared with normal adjacent tissues, the expression of PD-L1 in ESCC tissues was significantly increased (**Figure 4A**) (*P* < 0.001). In the cluster subtypes of cluster 1 and cluster 2 that we constructed, the expression difference of PD-L1 was not significant (**Figure 4B**). In addition, the expression of PD-L1 in ESCC patients was significantly negatively correlated with the expression levels of YTHDF2, METL14 and KIAA1429 (**Figure 4C**). The ratio of 22 immune cell types between the two subgroups was analysed (**Figure 5**).

### Immune Cell Infiltration in Consensus Cluster Subgroups of Esophageal Squamous Cell Carcinoma

**Figure 6:**
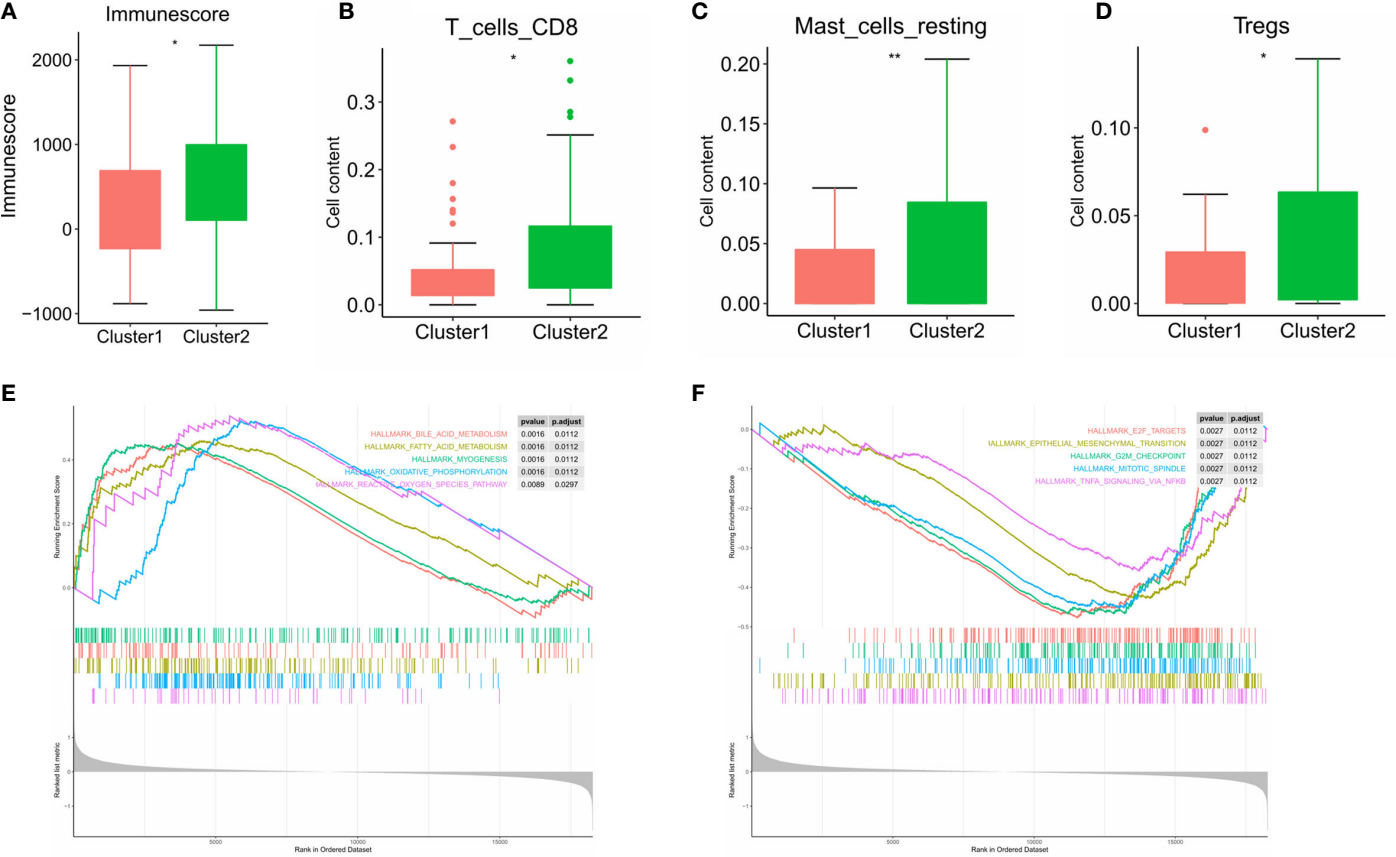
Differences in the level of immune cell infiltration between the two clusters in ESCC cohort and the biological pathways involved. **(A)** immune score in the cluster 1/2 subtypes; **(B–D)** the infiltrating levels of the CD8 T cells **(B)**, resting mast cells **(C)**, and regulatory T cell **(D)** in two clusters; **(E, F)** GSEA shows the first 5 signaling pathways involved in cluster 1 **(E)** and cluster 2 **(F)**. * means p<0.05; ** means p<0.01.

Subsequently, we evaluated the immune scores of the ESCC immune microenvironment based on the ESTIMATE algorithm and found that the immune scores of the two m6A RNA methylation-related clusters were significantly different (**Figure 6A**). Our research shows that cluster 2 exhibits high levels of CD8 T cells, resting mast cells and regulatory T cell (Treg) infiltration (**Figures 6B–D**) (*P* < 0.05). To explore the underlying regulatory mechanism that led to the TIME difference between the two subgroups, we performed GSEA. The results showed that the first 5 hallmark pathways that were significantly enriched in cluster 1 included bile acid metabolism, fatty acid metabolism, myogenesis, oxidative phosphorylation, and reactive oxygen species pathway, while the hallmark pathways involved in cluster 2 included E2F targets, the epithelial-mesenchymal transition, the G2/M checkpoint, the mitotic spindle and TNFA signalling *via* NFKB (**Figures 6E, F**).

### Construction of Prognostic Signatures Based on m6A RNA Methylation Genes

**Figure 7:**
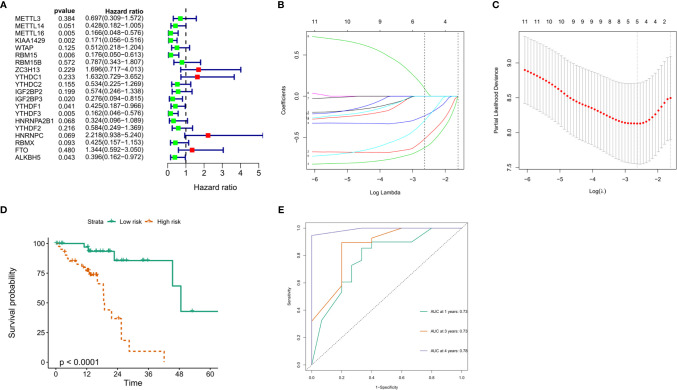
Construction of the prognostic signature based on TCGA ESCC cohort. **(A)** Univariate analysis of 20 m6A RNA methylation regulators to identify the genes that significantly correlated with OS; **(B, C)** The prognostic signature constructed by the minimum criterion of LASSO Cox regression algorithm; **(D)** The Kaplan-Meier curve shows that the risk score based on the prognostic signature of m6A RNA methylation is significantly correlated with OS in ESCC patients; **(E)** time-dependent ROC curves was applied to assess the predictive efficiency of the signature in TCGA.

To explore the prognostic value of these 20 m6A RNA methylation regulators in ESCC, we performed univariate Cox regression. The results demonstrated that METTL16 (*P* = 0.005), KIAA1429 (*P* = 0.002), RBM15 (*P* = 0.006), IGF2BP3 (*P* = 0.02), YTHDF1 (*P* = 0.041), YTHDF3 (*P* = 0.005) and ALKBH (*P* = 0.043) were significantly correlated with OS (**Figure 7A**). The hazard ratio (HR) of these genes with prognostic value was less than 1. Subsequently, the LASSO algorithm was used to obtain the coefficient of each prognostic gene (**Figures 7B, C**). According to the minimum standard, 5 m6A regulators (HNRNPC, RBM15, IGF2BP3, METTL16 and KIAA1429) were selected to construct a prognostic signature, and the risk score of each ESCC patient was calculated. The formula was as follows: risk score = (0.0933 * HNRNPC expression) − (0.2370 * RBM15 expression) − (0.2949 * IGF2BP3 expression) − (0.5176 * METTL16 expression) − (0.6240 * KIAA1429 expression). According to the median risk score, ESCC patients were divided into low-risk and high-risk groups.

### Risk Score Based on the Prognostic Signature Is an Independent Prognostic Factor in the ESCC Cohort of TCGA and GEO

**Figure 8:**
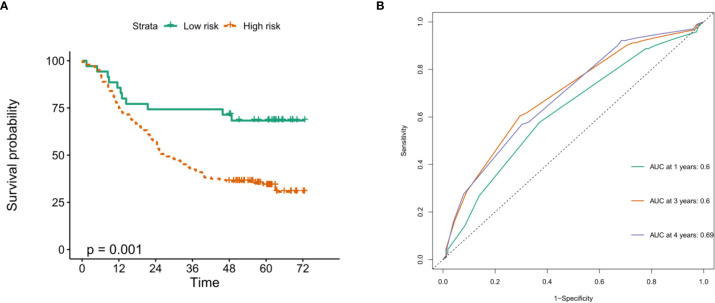
Verification of the prognostic value of m6A methylation regulator-related signatures in the GEO database. **(A)** The high risk score of the signature in the GEO database indicates poor OS; **(B)** time-dependent ROC curve in the GEO database confirms the predictive efficiency of the prognostic signature.

To verify the prognostic value of risk grouping in ESCC patients, Kaplan-Meier survival analysis was performed. The results showed that the OS of patients in the high-risk group was significantly lower than that in the low-risk group (**Figure 7D**) (*P* < 0.0001). Subsequently, ROC curves were drawn to evaluate the specificity and sensitivity of the prognostic signatures associated with m6A RNA methylation regulators. The results showed that the areas under the curve (AUCs) at 1, 3 and 5 years were 0.73, 0.73 and 0.78, respectively, indicating good prediction performance (**Figure 7E**). Importantly, we used the GSE53625 dataset from the GEO database to verify the prognostic value of the prognostic signature in ESCC. The results also showed that the high-risk score group was significantly related to worse prognosis in patients (**Figure 8A**). The 1-, 3-, and 4-year area under the curve (AUC) values of the ROC curve were 0.6, 0.6, and 0.69, respectively, exhibiting a good distinguishing performance for ESCC patients (**Figure 8B**).

**Figure 9:**
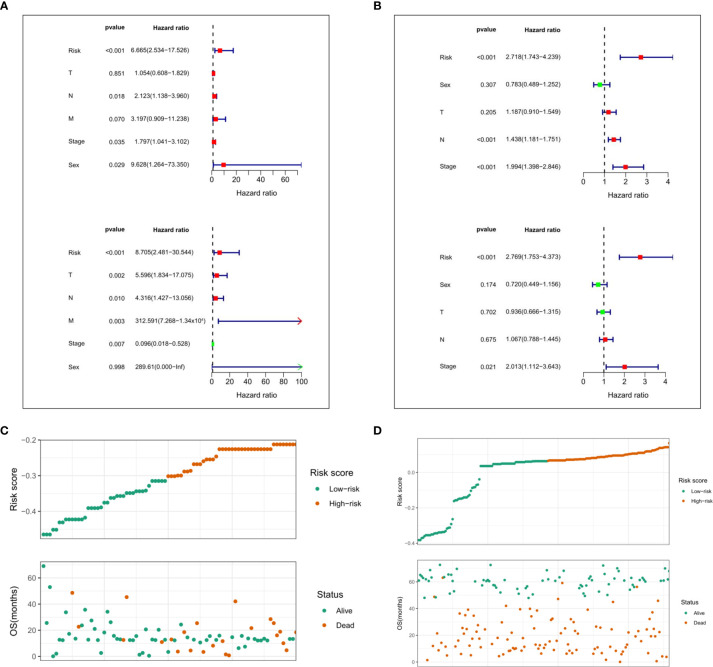
Evaluation of prognostic values of the risk scores. Univariate and multivariate Cox regression analysis of the risk scores in TCGA. **(A)** and GEO **(B)** database. The distributions of risk scores and OS status in TCGA **(C)** and GEO **(D)** database.

Moreover, we performed univariate and multivariate Cox regression analyses in the two cohorts of TCGA and GEO to determine whether the risk score based on prognostic markers is an independent prognostic indicator for ESCC patients. In the TCGA cohort, after univariate analysis obtained factors related to OS in ESCC patients, multivariate Cox regression analysis showed that risk score (*P*<0.001, HR = 6.665), N stage (*P* = 0.018, HR = 2.123), TNM stage (*P* = 0.035, HR = 1.797), and sex (*P* = 0.029, HR = 9.628) were identified as independent prognostic factors (**Figure 9A**). The risk scores, OS and OS status distributions of 95 ESCC patients from the TCGA database are shown in **Figure 9C**. The prognostic value of the risk score calculated with the prognostic signatures of 5 m6A RNA methylation modulators was verified in 358 ESCC patients from the GEO database. The univariate analysis showed that the risk score (P < 0.001, HR = 2.718), N stage (P < 0.001, HR = 1.438) and TNM stage (P < 0.001, HR = 1.994) were significantly correlated with OS, and subsequent multivariate Cox regression analysis demonstrated that the risk score (P < 0.001, HR = 2.769) and TNM stage (P = 0.021, HR = 2.013) were independent prognostic factors for ESCC patients (**Figure 9B**). The risk scores, OS and OS status distributions of ESCC patients verified in the GEO database are shown in **Figure 9D**.

### Prognostic Risk Scores Correlated With Stage, Immune Score, and Clinicopathological Parameters in ESCC

**Figure 10:**
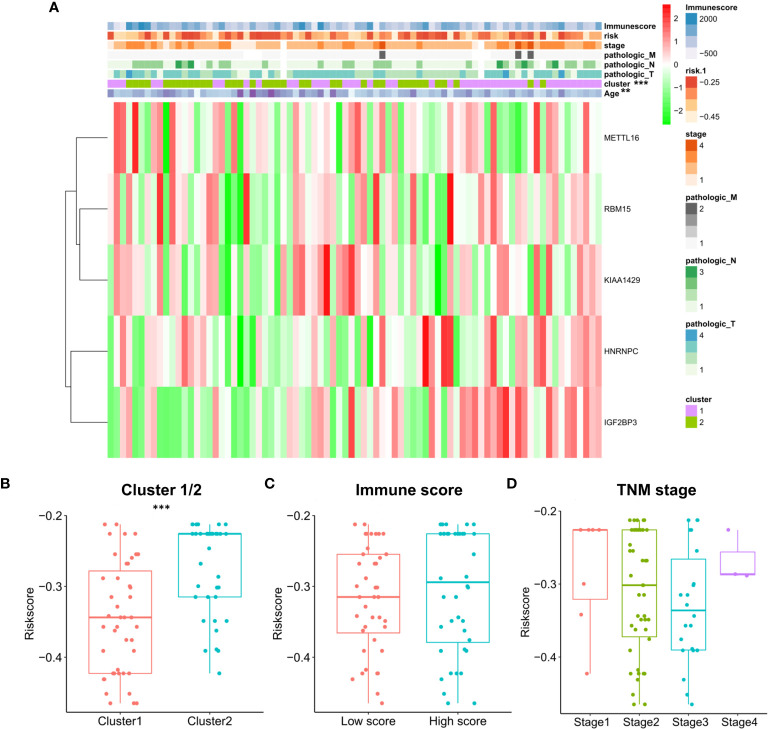
Prognostic risk scores correlated with stage, immune score, and clinicopathological parameters in ESCC. **(A)** Heatmap and clinicopathologic parameters of high and low risk groups; The relationship between risk score and cluster 1/2 **(B)**, immune score **(C)** and TNM staging **(D)**.

The relationship between risk score and clinical characteristics and cluster subgroups was further evaluated. It can be seen from the heatmap that the high immune score generally corresponds to the high expression of the 5 regulators. HNRNPC and IGF2BP3 are highly expressed in cluster 1. There were significant differences between the high-risk group and the low-risk group in clustering subtypes (P <0.001) and age (P <0.05) of ESCC (**Figure 10A**). We also further examined the relationship between risk score and subtype, immune score and TNM staging. The results show that the risk score of cluster 2 is significantly higher than that of cluster 1 (P <0.001, **Figure 10B**). Although the high and low immune scores and risk scores are not significant, it can be seen that the median risk score of the high immune score group is higher than that of the low immune score group (**Figure 10C**). In addition, TNM staging and risk score were not found to have significant statistical significance (**Figure 10D**). These findings indicate that the risk score of ESCC patients may profoundly influence clinical outcomes.

### Effect of Genetic Alterations of the m6A Regulator Signatures on Immune Cell Infiltration

**Figure 11:**
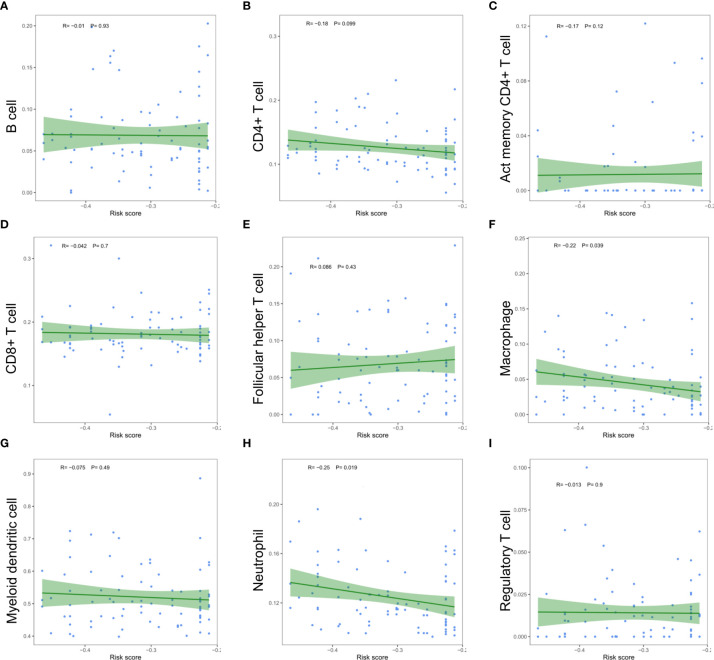
Relationships between the risk score and infiltration abundances of nine immune cell types. **(A)** B cell; **(B)** CD4+ T cell; **(C)** activated memory CD4+ T cell; **(D)** CD8+ T cell; **(E)** follicular helper T cell; **(F)** macrophage; **(G)** myeloid dendritic cell; **(H)** neutrophil; **(I)** regulatory T cell.

The relationship between the risk scores of nine immune cell types and the level of infiltration was analyzed to evaluate the impact of five m6A regulator-based signatures on the ESCC immune microenvironment. Perhaps due to the limitation of sample size, we only found a significant negative correlation between the risk score and the infiltration level of macrophages (P =0.039) and neutrophils (P =0.019, **Figure 11**). Risk signatures based on m6A regulators are likely to have a potential role in regulating the immune microenvironment of ESCC.

**Figure 12:**
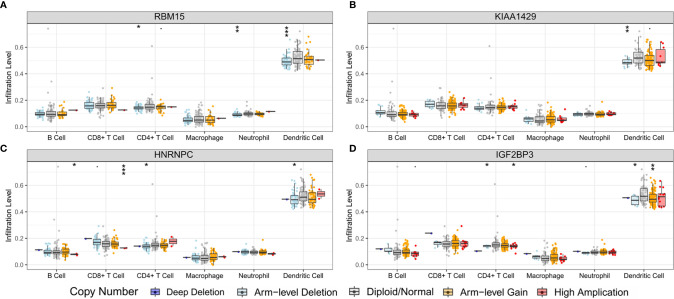
Effect of the genetic alterations of m6A regulator-relevant signature on the immune cell infiltration. **(A)** RBM15; **(B)** KIAA1429; **(C)** HNRNPC; **(D)** IGF2BP3. * means p<0.05; ** means p<0.01; *** means p<0.001.

In order to preliminarily clarify the potential mechanism of risk score and different immune cell infiltration, the influence of somatic cell CNAs based on m6A regulator on immune cell infiltration was further analyzed. The identified CNAs of m6A regulator signatures, including arm-level deletion, high amplication, and arm-level gain, significantly affected the infiltration level of B cells, CD4 + T cells, CD8 + T cells, neutrophils and dendritic cells in ESCC (**Figure 12**). This series of studies further show that m6A methylation regulators have a key regulatory effect on TIME in ESCC patients.

## Discussion

ESCC is a highly malignant tumor. Surgery combined with radiotherapy, chemotherapy, and targeted therapy are currently the main treatments for ESCC. However, due to the high rate of recurrence and metastasis, the survival results for this cancer are far from satisfactory (22–24). Although related immunotherapies involving esophageal cancer are still in the preliminary research stage, some related inhibitors have entered clinical trials and have shown long-lasting antitumor activity and controllable adverse reactions, which indicates that the immune microenvironment of ESCC warrants further exploration (7). At the same time, m6A methylation, which is the most common form of mRNA modification, has been proven to promote or suppress cancer in many tumor types (25), but there are very few related studies in ESCC. Therefore, it is necessary to further explore the role played by m6A methylation in ESCC and the infiltration of the ESCC TIME. In addition, the effect of m6A methylation on the ESCC TIME has not been fully elucidated.

In this study, the expression patterns, prognostic values and effects on the TIME of m6A RNA methylation regulators in ESCC were explored. The expression levels of m6A “writers” (METTL3 and WTAP) and “readers” (i.e., IGF2BP3, YTHDF1, HNRNPA2B1 and HNRNPC) in ESCC were significantly higher than those in normal tissues, and elevated expression levels of 2 genes (METTL3 and IGF2BP3) have been reported to be an independent prognostic factor for ESCC patients (26, 27); other genes have not been studied. In STRING, a protein-protein interaction (PPI) network composed of 20 m6A RNA methylation regulators was generated, and the biological functions involved in the regulators were preliminarily analysed through GO functional annotation. In addition, KIAA1429 and YTHDF3, which have the highest correlation coefficients, have not been reported in tumors, and they are likely to play important roles in the occurrence and development of ESCC.

Next, two molecular subtypes (clusters 1/2) were determined by consensus clustering of 20 m6A methylation regulators. Perhaps due to insufficient sample size, there was no significant difference between cluster 1/2 subtypes and clinicopathological parameters of ESCC patients. We also determined that the expression of PD-L1 in ESCC tumor tissues was significantly higher than that in normal tissues. This phenomenon is not obvious in the cluster 1/2 subtype. In addition, it has been observed that PD-L1 is significantly negatively correlated with YTHDF2, METL14, and KIAA1429. These regulators are likely to predict the efficacy of immunotherapy in ESCC patients, which requires further research. We also studied the immune cell infiltration of the TIME in the cluster 1/2 subtype based on m6A regulators in ESCC patients. The results showed that the infiltration level of CD8 T cells, resting mast cells and regulatory T cells (Tregs) in cluster 2 was higher than that in cluster 1. Studies have shown that high mast cell density is related to the progression of ESCC and reduced postoperative survival. The high concentration of Tregs in ESCC can lead to immune escape and promote tumor progression (28, 29). The immune score calculated based on the ESTIMATE algorithm is also significantly higher in cluster 2, which indicates a significant difference in the TIME of ESCC patients. GSEA of cluster 1 was performed to explore the biological processes involved, and the results showed that it involved multiple carcinogenic pathways of digestive tract tumors (30–33). Similarly, we determined the signaling pathway involved in the regulator in cluster 2, and the relationship between it and the level of immune cell infiltration in the tumor microenvironment warrants further exploration.

Furthermore, we also constructed a five-gene prognostic signature consisting of HNRNPC, RBM15, IGF2BP3, METTL16, and KIAA14297 from m6A methylation regulators, and the calculated risk score showed good performance in predicting the prognostic outcome of ESCC patients. In addition to the risk score, N stage, TNM stage and sex were also independent prognostic factors. Importantly, the potential of m6A methylation modulator-related prognostic signatures was verified in another ESCC cohort from the GEO database. The GSE53625 verification cohort of 358 patients also shows the strong prognostic potential of this signature. A high risk score is significantly related to poor OS in ESCC patients and is an independent prognostic factor for ESCC patients. Among these risk markers, HNRNPC can interact with LBX2-AS1 and enhance the stability of ZEB1 and ZEB2 mRNA, thereby promoting the migration and development of ESCC (34). Another study showed that the high expression of HNRNPC is significantly related to the poor OS of patients with lung adenocarcinoma, and it is likely to be an oncogene in patients with lung adenocarcinoma and breast cancer (35, 36). IGF2BP3 is highly expressed in many tumors, including ESCC, lung adenocarcinoma, colon cancer, and gastric cancer, and leads to poor prognosis (26, 37–39). There is no research report on KIAA1429 in ESCC, but some scholars pointed out that KIAA1429 can promote the progression of liver cancer, breast cancer and osteosarcoma, leading to poor prognosis (40–42). For RBM15 and METL16, there are currently few reports in tumors, but they may be potential prognostic biomarkers.

At present, the effect of m6A methylation regulators on immune cell infiltration in TIME is still unclear. In this study, the risk score based on the prognostic signatures of five m6A regulators was significantly negatively correlated with the infiltration level of macrophages and neutrophils. High immune scores often correspond to high expression of HNRNPC and IGF2BP3. Studies have shown that HNRNPC and HNRNPK in the subfamily of heterogeneous ribonucleoproteins (hnRNPs) can regulate the recruitment and activation of neutrophils and macrophages respectively, thereby affecting the systemic immune response (43, 44). The research on the other three risk signatures and immune cell infiltration remains unclear. For other m6A regulators, Han et al. showed that in mouse tumors lacking YTHDF1, the level of CD8 + T and NK cell infiltration increased, thereby enhancing the cross-expression of tumor antigens *in vivo* and the cross-priming of CD8 + T cells (17). Li et al. reported that the loss of METT3 or METT14 triggers the disorder of T cell proliferation and differentiation, thereby reducing the sensitivity of interleukin 7 (IL-7) *in vivo* (45). Our study also showed that CNAs of m6A methylation regulators, including arm level deletion, high amplication and arm level gain, significantly affect the level of immune cell infiltration in ESCC. The m6A methylation regulator is likely to have a crucial regulatory effect on TIME in ESCC patients.

This study has several limitations. First, the results may be affected by the small sample size. It is necessary to further improve the sample size, sequencing data and clinical information of ESCC patients in future research. In addition, our conclusions are based on the results of bioinformatic analysis of datasets containing genetic and other molecular information from patient tissues, which need to be further verified in clinical studies.

In summary, this study systematically evaluated the expression of m6A RNA regulators in ESCC, their correlation with PD-L1, and potential regulatory mechanisms. Two ESCC subtypes (clusters 1/2) were obtained through consensus clustering of m6A regulators, and the difference in the level of immune cell infiltration in the TIME was determined. m6A RNA regulators may improve the responsiveness of ESCC patients to immunotherapy by regulating the TIME and expression of PD-L1. More importantly, we constructed a prognostic signature containing 5 genes based on m6A RNA methylation, and the risk score was determined to be an independent prognostic factor in two different ESCC cohorts, indicating that the prognostic signature is a promising tool for predicting the survival outcome of ESCC patients.

## Data Availability Statement

The original contributions presented in the study are included in the article/supplementary material. Further inquiries can be directed to the corresponding authors.

## Author Contributions

JH, SG, WG, and FT conceived, designed, and supervised the study. WG, FT, and QH drafted the manuscript. WG, FT, QH, ZW, FS, GZ, ZY, RL, and QX collected the data. QH, ZW, FS, GZ, ZY, RL, and QX performed all data analysis. All authors contributed to the article and approved the submitted version.

## Funding

This work was supported by National Key R&D Program (2018YFC1312105), the Institutional Fundamental Research Funds (2018PT32033), the Ministry of Education Innovation Team Development Project (IRT-17R10), and the Beijing Hope Run Special Fund of Cancer Foundation of China (LC2019B15).

## Conflict of Interest

The authors declare that the research was conducted in the absence of any commercial or financial relationships that could be construed as a potential conflict of interest.
